# Producing an evidence‐based treatment information website in partnership with people affected by multiple sclerosis

**DOI:** 10.1002/hsr2.24

**Published:** 2018-03-06

**Authors:** Anneliese J. Synnot, Melanie Hawkins, Bronwen A. Merner, Michael P. Summers, Graziella Filippini, Richard H. Osborne, Sue D.P. Shapland, Catherine L. Cherry, Rwth Stuckey, Catherine A. Milne, Paola Mosconi, Cinzia Colombo, Sophie J. Hill

**Affiliations:** ^1^ Centre for Health Communication and Participation, School of Psychology and Public Health La Trobe University Melbourne Australia; ^2^ Cochrane Australia, School of Public Health and Preventive Medicine Monash University Melbourne Australia; ^3^ Health Systems Improvement Unit, Centre for Population Health Research, School of Health and Social Development Deakin University Geelong Australia; ^4^ Cochrane Multiple Sclerosis and Rare Diseases of the Central Nervous System Review Group, Scientific Direction IRCCS Foundation Neurological Institute Carlo Besta Milan Italy; ^5^ MS Australia Perth Australia; ^6^ Burnet Institute, Department of Infectious Diseases The Alfred Hospital and Monash University Melbourne Australia; ^7^ Faculty of Health Sciences University of the Witwatersrand Johannesburg South Africa; ^8^ Centre for Ergonomics and Human Factors, School of Psychology and Public Health La Trobe University Melbourne Australia; ^9^ Centre for Values, Ethics and Law in Medicine University of Sydney Sydney New South Wales Australia; ^10^ Laboratory for Medical Research and Consumer Involvement, Department of Public Health IRCCS Istituto di Ricerche Farmacologiche Mario Negri Milan Italy

**Keywords:** Cochrane, consumer participation, evidence‐based treatment information, health education, multiple sclerosis, patient and public involvement, systematic review

## Abstract

**Background and Aims:**

In earlier work, we identified that people affected by multiple sclerosis (MS) can have difficulty finding online treatment information that is up to date, trustworthy, understandable, and applicable to personal circumstances, but does not provoke confusion or negative emotional consequences. The objective was to develop online consumer summaries of MS treatment evidence (derived from Cochrane Reviews) that respond to identified treatment information needs of people affected by MS.

**Methods:**

A 2‐phase mixed‐methods project, conducted in partnership with consumers and an MS organisation. Phase 1 included review panels with consumers (Australians affected by MS) and health professionals to test paper‐based treatment summaries before development, and pilot testing of the website. Phase 2 involved an online survey after website launch.

**Results:**

Eighty‐three participants (85% affected by MS) took part. Phase 1 participants strongly endorsed key review summary components, including layering information, and additional sections to aid personal applicability. Participants additionally suggested questions for health professionals. Participants across both phases were receptive to the idea of being provided with Cochrane Review summaries online but were seeking other types of evidence and information, such as personal experiences and the latest experimental treatments, which could not be provided. While the small survey sample size (n = 58) limits application of the results to a broader population, the website was viewed favourably, as a useful, understandable, and trustworthy information source.

**Conclusion:**

We describe a partnership approach to developing online evidence‐based treatment information, underpinned by an in‐depth understanding of consumers' information needs.

## Abstract 2

Background and Aims

In earlier work, we identified that people affected by multiple sclerosis (MS) can have difficulty finding online treatment information that is up to date, trustworthy, understandable, and applicable to personal circumstances, but does not provoke confusion or negative emotional consequences. The objective was to develop online consumer summaries of MS treatment evidence (derived from Cochrane Reviews) that respond to identified treatment information needs of people affected by MS.

Methods

A 2‐phase mixed‐methods project, conducted in partnership with consumers and an MS organisation. Phase 1 included review panels with consumers (Australians affected by MS) and health professionals to test paper‐based treatment summaries before development, and pilot testing of the website. Phase 2 involved an online survey after website launch.

Results

Eighty‐three participants (85% affected by MS) took part. Phase 1 participants strongly endorsed key review summary components, including layering information, and additional sections to aid personal applicability. Participants additionally suggested questions for health professionals. Participants across both phases were receptive to the idea of being provided with Cochrane Review summaries online but were seeking other types of evidence and information, such as personal experiences and the latest experimental treatments, which could not be provided. While the small survey sample size (n = 58) limits application of the results to a broader population, the website was viewed favourably, as a useful, understandable, and trustworthy information source.

Conclusion

We describe a partnership approach to developing online evidence‐based treatment information, underpinned by an in‐depth understanding of consumers' information needs.

## 1 BACKGROUND

Multiple sclerosis (MS) is a chronic neuro‐inflammatory condition with much uncertainty around individual prognosis. Treatments include a diverse array of drugs with some confronting risk‐benefit profiles^1^ and many novel and experimental treatments.^2^ Like many health care consumers, people affected by MS, that is, people with MS and their families and carers, increasingly seek information about such treatments online.^3^, ^4^ While most trust the advice of their health professionals,^3^ many people affected by MS gain confidence over time to find and interpret online treatment information.^5^

Within this context, it is important to provide people affected by MS with up‐to‐date, reliable, and independent evidence‐based treatment information. Systematic reviews, as summaries of multiple studies on a topic, should be the basis for much of the information about benefits and harms.^6^ However, previous research has found that consumers, that is, patients, families, carers, and their advocates, can find systematic reviews, and plain language summaries, either difficult to understand or insufficiently detailed to use in decision making.^7^, ^8^ They also want to know how research evidence relates to them individually, and determining this can be difficult.^7^, ^9^ While the evidence is building on how to best share systematic review evidence with consumers,^7^, ^9^, ^10^ including people affected by MS,^11^, ^12^ until recently, there has been scant literature or examples about how this translates into an online environment within the context of MS^13^ and how to integrate systematic review data with other information needs.^9^ Recent developments in consumer‐friendly online presentations of benefits and harms include interactive Summary of Findings tables and plain language summaries^14^ and the MAGICApp rapid recommendations and decision aids.^15^

This paper describes the development of *Making Sense of MS Research*,^16^ an Australian website launched in 2012, that provides summaries of MS treatment evidence from Cochrane Reviews for people affected by MS. We created the website as part of “Integrating and Deriving Evidence, Experiences and Preferences” (IN‐DEEP), a mixed‐methods study conducted in parallel in Australia^5^, ^17^ and Italy.^18^, ^19^ Here, we describe the Australian arm of the project.

For the first stage of IN‐DEEP, we undertook a qualitative exploration of the online treatment information‐seeking experiences, preferences, and needs of 60 Australians with MS and their family members.^5^ We identified that people affected by MS can have difficulty finding online treatment information that is up to date, trustworthy, understandable, and applicable to personal circumstances, but does not provoke confusion or negative emotional consequences.

In stage 2, described here, we sought to respond to the unmet treatment information needs identified in stage 1, by developing *Making Sense of MS Research*.^16^ As such, the aim of this project stage was to develop online consumer summaries of MS treatment that were easy to understand and applicable to personal circumstances, met additional information needs, relied on up‐to‐date and trustworthy sources, and mitigated confusion and negative emotional consequences.

## 2 METHODS

The methods for this project were informed by the principles of knowledge translation^6^ and consumer participation in research.^20^ In knowledge translation, the focus is not just on the content and message being shared but is also founded on an understanding of the target audience and the broader context.^21^ Involving consumers in research has an intrinsic value of encouraging democracy, accountability, and transparency and an extrinsic value of improving the quality, impact, and relevance of the research produced.^22^, ^23^ In practice, both involve interactive processes that share research between its creators and users, with varied activities and multiple iterations.^6^

We describe this study in 2 phases. Phase 1—developing the website—involved the development of paper‐based treatment summaries that were tested in review panels involving people affected by MS and others, followed by website creation and pilot testing. The purpose of these review panels and website pilot testing was to help inform the website content and design the information presentation format. Phase 2—website feedback survey—occurred after the website was launched and involved an online survey of website visitors. The purpose of the evaluation of the consumer summaries and the website was to gather user feedback about the presentation and usefulness of the consumer summaries and about website navigation. Reflecting commonly used terminology in Australia, in this paper, we use the term “consumer,” which means patients, families, carers, their advocates, and representatives, interchangeably with “people affected by MS.”

### 2.1 Project team, and consumer and operational advisory groups

The project team included Australian (SH, AS, MH, and RO) and Italian researchers (GF, CC, and PM), and staff from Australian and local (Victorian) MS societies (MPS and SS). We also sought input from 2 advisory groups convened for the project: a consumer advisory group and an operational advisory group. The consumer advisory group (CLC, RS, and CAM) included 2 people with MS and an MS health information specialist. They prioritised the Cochrane Reviews to be included on the website and provided advice on data collection and analysis, and input into website layout, content, and wording.^24^ The operational advisory group included clinical managers (n = 2), IT staff (n = 2), and communications personnel (n = 2) from the local Australian state‐based MS organisation. They played a major role in the design and technical aspects of the website and reviewed content from a clinical perspective.

### 2.2 Phase 1: developing the website

The development of the treatment summaries from the Cochrane Reviews, along with all additional website content, and overarching structure and format, was informed by existing literature and best practice documents. There were also shaped by the findings of the previous IN‐DEEP study (stage 1),^5^ together with input from people with MS, and others, described in here (IN‐DEEP stage 2).

#### 2.2.1 Developing paper‐based summaries

We selected 2 Cochrane Reviews of MS treatments (prioritised by the consumer advisory group) and developed them into paper‐based treatment summaries for feedback by the review panels. We chose these reviews (one on a medication used by many people affected by MS^25^ and the other about exercise^26^) because of the relevance of this information to many people and because of the different types of data to be summarised. We followed research‐based principles for presenting treatment information to consumers, such as layering,^27^ and used a combination of words, numbers and pictures, and absolute, rather than relative, frequencies.^28^, ^29^ The information we presented differed from the usual format of a Cochrane Review in the following ways: It was significantly shorter and focused on key information only (ie, inclusion criteria and results); plain language was used throughout; graphical illustrations of numerical information were used; and novel sections, explaining how results applied to individuals and questions that consumers can ask their health professionals, were included.

#### 2.2.2 Review panels

The aim of the review panels was to seek formal feedback on the paper‐based treatment summaries before they were transferred to an online format. Recruitment involved purposive sampling from participants of the first stage of the IN‐DEEP project^5^ and from the networks of the project team and the MS organisation. We sought to include people affected by MS, clinicians, and staff of the MS organisation.

One week prior to the review panels, we mailed participants a pack containing a document explaining the nature of the project, the 2 treatment summary templates, and information and consent forms. We included a one‐page feedback form, whereby participants were encouraged to record their immediate impressions of the templates and bring this to the panel discussion.

We held 2 review panels (a form of group interview)^30^, ^31^ at a local MS organisation office, with each panel session lasting 2 hours. Two researchers were involved as facilitator (SH) and note‐taker (AS). The following 5 questions, based on those used in the DECIDE project (work package one),^32^ were used to guide the discussion: “do you understand the information presented?”; “is it helpful to you?”; “is there anything missing?”; “is there anything superfluous?”; and “do you have any suggestions for improvement?”

One researcher (AS) collated participants' feedback sheets, the researchers' detailed written notes, and audio recordings of the review panel sessions, which were reviewed by a second researcher (SH). We used the audio recordings to supplement the written notes. One researcher (AS) grouped all participant feedback under 1 of 5 categories: one overarching category on content, formatting, or general feedback, and 4 specific categories corresponding to the 4 main sections of the treatment summary template (ie, “The short answer,” “The detailed answer,” “What does this mean for me?” and “Using this information”). Within each of these categories, individual feedback items were grouped according to “likes,” “dislikes,” and “suggestions.” We considered the implications of each individual feedback item, culminating in a list of recommendations for changes and/or additions to the individual review templates or, more broadly, for the website. We subsequently discussed and agreed on the recommendations with the project team and consumer advisory group.

#### 2.2.3 Developing the pilot website

We created a pilot website after integrating feedback from the review panels. To guide the website layout and format, we used the HONcode principles^33^ and the Harvard School of Public Health Guidelines.^34^ Web design and functionality were informed by the Web Accessibility Initiative Guidelines,^35^ as well as by input from the consumer advisory group. To cater for people who did not have access to the Internet and to enable website visitors to discuss the treatment summaries with their health professional, we created downloadable PDF versions of the treatment summaries. MS organisation clinical managers and an MS organisation librarian reviewed the content for relevance to the Australian context and for readability.

#### 2.2.4 Website pilot testing

After the development of the pilot website, we invited people affected by MS to take part in pilot testing. Participants were recruited from people who took part in the review panels and through the networks of the project team.

We asked pilot testing participants to visit the website and complete an online survey (see Supporting Information). Participants subsequently took part in a 30‐minute telephone interview to discuss their survey responses with a researcher who had not been involved in the development of the website (MH). In response to the survey data and interview responses, we made minor changes to the website, such as incorporating additional links to further information and reordering of the site page tabs.

### 2.3 Phase 2: website feedback survey

Following incorporation of the changes after pilot testing, we launched the website in November 2012, with promotion to relevant organisations and individuals across Australia and internationally. The primary promotion strategy was through the networks of the local MS organisation and MS Australia, via their websites and social media pages, e‐mailing lists, and newsletters.

#### 2.3.1 Online survey

The purpose of the evaluation of the consumer summaries and the website was to gather user feedback about the presentation and usefulness of the consumer summaries and about website navigation. Development of the survey was based on the unmet treatment information needs identified in stage 1 of IN‐DEEP.^5^ On the basis of these unmet needs, we developed survey sections with brief descriptive rationales for survey questions. Two project team members (RO and MH) developed the survey questions using a rapid literature review and their extensive survey‐writing experiences and resources. The questions chosen for survey sections were informed by previously validated patient‐reported outcomes measures.^36^, ^37^ Collaboration with the research team about survey content was ongoing. The final survey included qualitative and quantitative questions under sections about users' initial expectations of the site and what else they would like to see on it; how the information was presented on the website and if users might use the information to consult health professionals; the site's usability; and demographic questions. The survey was pilot tested with people affected by MS as part of the broader website pilot testing, and some minor revisions were made to improve user understanding of the questions. This survey was designed for the purpose of gathering user feedback about presentation of the online consumer summaries and website usability and was not intended for use in other contexts. We included the survey on the website for 2 months after the site was launched (see Supporting Information) but did not actively recruit people to complete the survey. It displayed as a box of highlighted text that website users could accept or decline.

### 2.4 Ethical approval and informed consent

Participants in all phases provided informed consent to participate, via a written or online form. We received approval from the Human Ethics Committee, Faculty of Health Sciences, La Trobe University (numbers 11‐169 and 12‐128). We offered review panel participants a $50 voucher for their participation. We did not offer reimbursement to participants in other project phases.

## 3 RESULTS

### 3.1 Participants

A total of 83 participants took part of the project, participating in review panels (n = 16), website pilot testing (n = 9), or by completing the website feedback survey (n = 58) (see Table 1). Of the 61 participants who provided demographic information, the majority were people with MS (75%) or family members of people with MS (12%). Over two‐thirds of participants were female (71%) and aged between 41 and 65 years (73%). All but one participant resided in Australia. Approximately half of the participants affected by MS had a university degree (48%), although those in the final project stage (website feedback survey) included a broader range of educational backgrounds, with 34% of respondents having an educational level of high school or below. The median length of time since MS diagnosis was 8 years (range < 1 to 30 years), and most participants (69%) had the relapsing‐remitting form of the disease.

**Table 1:** Participant demographic characteristics

Characteristics	Developing the Website		Website Feedback Survey	Total (N = 83)
	Review Panels (N = 16)	Pilot Test (N = 9)	Online Survey (N = 58)^a^	
Participants (n, %)				
People with MS	10 (63)	6 (67)	30 (83)	46 (75)
Family members	1 (6)	3 (33)	3 (8)	7 (12)
Health professionals/other^b^	5 (31)	0 (0)	3 (8)	8 (13)
Gender (n, % female)	10 (63)	7 (78)	22 (73)	39 (71)
Age (n, %)				
21 to 40 y	3 (19)	3 (33)	6 (20)	12 (22)
41 to 65 y	11 (69)	6 (67)	23 (77)	40 (73)
66 y and over	2 (13)	0 (0)	1 (3)	3 (6)
Highest education level^c^ (n, %)				
High school (not completed)	0 (0)	0 (0)	5 (17)	5 (10)
High school (completed)	1 (9)	1 (11)	5 (17)	7 (14)
Occupational certificate	6 (55)	4 (44)	4 (13)	14 (28)
University degree	4 (36)	4 (44)	16 (53)	24 (48)
Time with MS^e^ (y; median, range)	19 (2 to 24)	4.5 (1 to 21)	4 (<1 to 30)	8 (<1 to 30)
Type of MS (n, %)^d^^,^ e				
RRMS	NA	5 (83)	20 (67)	25 (69)
PPMS	NA	1 (17)	5 (17)	6 (17)
SPMS	NA	0 (0)	3 (10)	3 (8)
Other/unsure	NA	0 (0)	2 (7)	2 (6)

Abbreviations: MS = multiple sclerosis; N, total number of participants; NA, not available; PPMS = primary progressive multiple sclerosis; RRMA; relapsing remitting multiple sclerosis; SPMS, secondary progressive multiple sclerosis.

a Twenty‐two participants did not provide full demographic details, so none of the total number of participants for each demographic characteristics adds up to 58.

b Included neurologists, MS nurses, and MS organisation staff.

c People with MS and family members only.

d People with MS only.

e Not asked of people with MS in the review panels.

### 3.2 Phase 1: developing the website

We present the results of this phase grouped under the 6 aims of the website.

#### 3.2.1 Easy to understand

**Figure 1:**
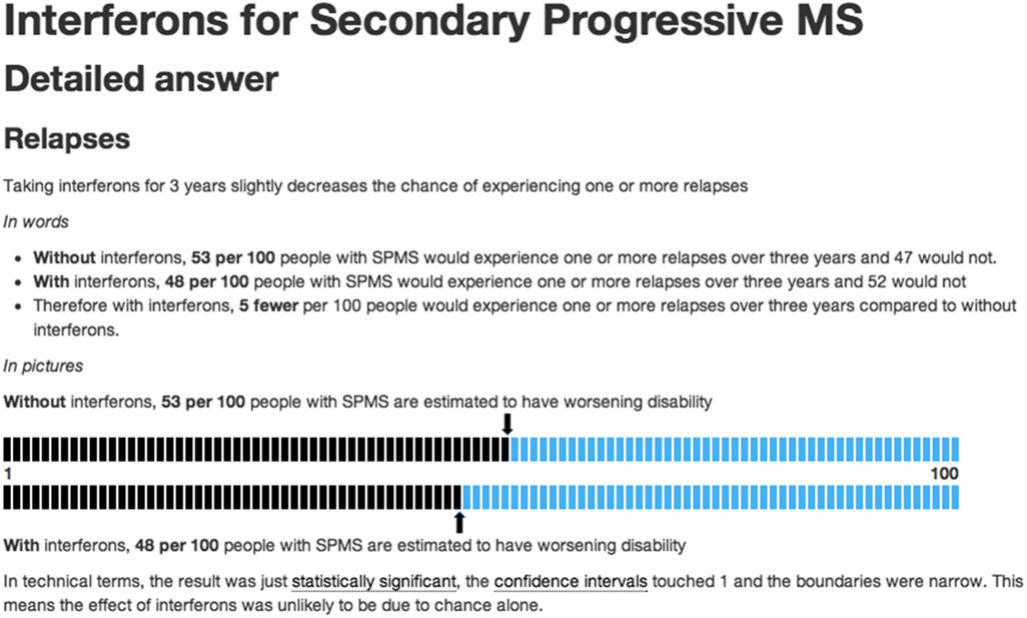
Screenshot showing presentation of the evidence in “the detailed answer”

To present the treatment information in a format that was straightforward and clear, we created 2 sections (the “short answer” and the “detailed answer”) to summarise the benefits and harms of the treatment, so that the information was effectively layered.^27^ In “the short answer” section, we provided a one‐paragraph summary of the Cochrane Review results. In “the detailed answer” section, a combination of words, numbers, and graphical images was used to describe the effect of the intervention compared with placebo, on the main outcomes collected in each review, thereby presenting the same review findings in 3 different formats^28^, ^29^ (see Figure 1). We expressed absolute numbers as natural frequencies (ie, 5 of 100).

Review panel participants endorsed the concept of layering the information and providing detailed scientific information in a combination of formats. Some of the nonconsumer participants were concerned about consumers' abilities to make sense of the information, but this was not reflected by consumer participants. There were mixed responses to the 100 smiley faces graphic that we used in the review panel sessions (see Kasper's multifigure pictographs^12^ for an example). After consultation with colleagues, a new horizontal comparison graphic was used on the website instead (see Figure 1). Several participants suggested that the addition of a hyperlinked glossary would be preferable to trying to explain scientific terms in the text. The glossary was subsequently created for the website.

#### 3.2.2 Applicable to personal circumstances

To provide treatment information that was more personally applicable, we created an additional section, specific to each treatment summary, called “Does this apply to me?” There, we described the characteristics of the people included in the studies and the parameters of the interventions studied therein.^9^ We also added a section called “Questions for my health professional” that was specific to each review.^33^ We developed a set of questions, in consultation with the consumer advisory group, that consumers could use as a prompt for discussion with their health professionals. Example questions included “How soon after diagnosis is it recommended to take interferons?” and “What kind of rehabilitation would be right for me?” Both the “Does this apply to me?” and “Questions for my health professional” sections were strongly endorsed in the review panels as ways to help readers apply the information to their specific situation.

#### 3.2.3 Meets additional information needs

To acknowledge the additional information needs of consumers, we created a section called “Find out more,” with the aim of connecting readers to their local MS organisation and providing links to other online information and evidence sources (selected in consultation with MS organisation staff).^9^, ^33^ This was one of the most strongly endorsed concepts in the review panels and the pilot testing, but many consumers wanted more information and links than those provided. Some review panel participants wanted links to patient websites where they could read about “real people's” responses to treatments. Others wanted more detailed information about interventions, such as what specific types of exercise (eg, walking or gym classes) one might look at starting or practical tips on how the medications were administered.

While the scope of the project was limited to treatment information only, we provided links to MS organisation resources and created a new section called “New to MS?” with some brief information about MS and a range of web links for further information. In pilot testing, we found that participants were expecting more treatment topics on the website. To address this, we created a poll on the website with 5 additional review topics and invited website visitors to vote for the topic to be summarised next. Visitors could leave their email address if they wished to be notified when new treatment summaries were added to the website.

#### 3.2.4 Trustworthy

At the review panels, there were questions about Cochrane and how Cochrane Reviews were created. For example, some participants asked if Cochrane was trustworthy and if the information was up to date. In response to this, we created a *Frequently Asked Questions* page, drawn from some of the questions posed at the review panels (for example, “What is the Cochrane Collaboration?” and “Why don't the treatment summaries always give a clear answer?”), and a page called “About the Research,” which explained the website development process. In addition, logos from the project partners of La Trobe University, the MS organisation, and Cochrane were included in the footer of every page to make clear the independence of the website creators from industry influence.^33^

#### 3.2.5 Up to date

To ensure website visitors would know the information was up to date, we included the date of the review publication and the search dates for each Cochrane Review that was summarised.^33^ Review panel participants endorsed the inclusion of these dates but queried the accuracy of the treatment information because the reviews were published more than 2 years earlier. As such, in addition to the information about the date of publication, we included a statement about whether or not newer studies had been published since that time. We only included Cochrane Reviews on the site when we were confident that no further trials had been published that met the inclusion criteria for the review (as advised by the Cochrane Multiple Sclerosis and Rare Diseases of the Central Nervous System Review Group).

To align available Cochrane Review topics with consumer preferences, we asked the consumer advisory group to prioritise the reviews to be included on the website. The prioritisation criteria included the expected interest to people affected by MS and how current the review was. Unfortunately, the Cochrane Reviews on topics of broad interest such as exercise,^26^ diet,^38^ and vitamin D supplementation^39^ were not, at that time, sufficiently up to date to be included. The final reviews selected for the website were on immunotherapies for different types of MS,^25^, ^40^, ^41^, ^42^ hyperbaric oxygen therapy,^43^ and rehabilitation.^44^

#### 3.2.6 Mitigates confusion and negative emotional consequences

To mitigate confusion or unnecessarily negative emotional consequences, consumer advisory group members reviewed the language used in the summaries, to ensure that it was appropriate to a broad range of consumers. We also added a question to the *Frequently Asked Questions* section to offer reasons as to why the review summaries could not always provide clear answers. To mitigate the risk of confusion or concern for website readers, we included questions such as “Why do these numbers look different from what I have seen elsewhere?” The website encouraged readers to contact their local MS organisation if they were concerned about conflicting information.

### 3.3 Phase 2: website feedback survey

Once launched, the website received 3873 visits (75% of which were unique visits) in the 2‐month period during which the feedback survey was live. During this evaluation period, 61 people elected to respond to the survey, with 58 useable responses included in the analysis (responses from 3 people were too incomplete to include). Given we did not actively recruit participants to the survey, but instead recruited them to view the website, the small sample size is not unexpected but does limit interpretation of the usefulness of the site to a broader population. However, of those who completed the survey, nearly 80% (n = 46) responded positively to the research summaries and the website as a whole. As well as quantitative data, respondents gave free‐text responses that indicated they found the information and website layout to be clear, aesthetically pleasing, and easy to navigate and understand. Overall, the presentation of the benefits and harms of each intervention was well received, with more than 75% of respondents agreeing or strongly agreeing with questions about understanding benefits and risks (see Supporting Information, S2 Table).

Other questions were designed to probe reflections about personal applicability (“The website has helped me to understand how the treatments might be useful for me”), trustworthiness (“The Cochrane Collaboration is a trustworthy source of information about treatments for health conditions”), meeting additional information needs (“I feel the About the Research section helped me to understand the summaries about MS treatments”), and emotional consequences (“I find it frightening to read about the risks of these treatments”). These were rated as “agree” or “strongly agree” by approximately two‐thirds (61%‐71%) of participants.

The main reasons for dissatisfaction with the information in the summaries were that they did not cover the latest approved and experimental MS medications and treatments, or nonmedical treatments. Questions with the lowest endorsement were about whether or not participants now felt able to make an educated decision about the treatments (21%), if the website had helped them understand how the treatments might be useful for them (19%), if the website met their information needs (17%), and if they found the “Does this apply to me?” section useful (17%).

## 4 DISCUSSION

In this study, we sought to address the unmet treatment information needs of people affected by MS, as established by phase 1 of this project, by developing a website based on MS Cochrane Review summaries. We undertook all project stages in partnership with people affected by MS and with the local MS society.^24^ As a consequence of this stakeholder consultation, the website was underpinned by an in‐depth understanding of treatment information–seeking experiences of people affected by MS. Responses across all project phases indicated there was a general receptivity by people affected by MS to the concept of having online, independent, evidence‐based treatment information. The website feedback survey responses, while modest in number, suggest the information was understandable, viewed as trustworthy, and addressed how people might apply the information to themselves. While the treatment summaries included information that was up to date, in that is was recent evidence, the feedback across all project stages and phases was that respondents wanted us to incorporate evidence about the latest, and sometimes experimental, treatments, which were not available as Cochrane Reviews at the time of this research.

While Cochrane Reviews remain the gold standard in systematic reviews of treatment research,^45^ and systematic reviews should form the basis of evidence‐based treatment information shared with consumers,^6^ they are insufficient for a complete treatment information website. Consumers also need general information about the disease and practical information, such as how treatments are administered.^46^ Additional challenges of using Cochrane Reviews are that they can only provide an answer if there is sufficient existing trial evidence and, like all systematic reviews, can date quickly in fast‐moving fields.^47^ This makes it particularly challenging to provide up‐to‐date information that includes coverage of new or experimental treatments on such a website. Originally, our treatment summaries were intended to be incorporated on our MS partner's website (where much of this additional information is provided). However, due to unforeseen circumstances during the project, we created a stand‐alone website to house the summaries. So, while we provided links to local MS organisations for further information, it was beyond the project scope and resources to provide all this additional information.

Similarly, the inclusion of personal narratives, or stories of the illness experiences of others in evidence‐based websites, is an emerging issue for health information providers. Echoing our results, other researchers have found that consumers want to know about the personal experiences of people with health conditions,^48^, ^49^ but there is uncertainty among the research community about how these experiences can be incorporated into evidence‐based resources. Personal narratives do affect people's judgment and choices, but how to harness this to facilitate, as opposed to hinder, patients' decision making is unclear.^50^ The use of scientifically rigorous methods to source and summarise personal narratives, such as those underpinning the Italian IN‐DEEP website^51^ and the HealthTalk website,^52^ offers, at least, a partial solution to this problem.

Our findings affirm the importance of partnerships between evidence producers and trusted information providers, such as peak bodies and member organisations. Not only do such organisations provide some of the additional information that consumers seek, but also groups like MS societies are considered trustworthy sources of health information.^3^ This is important within the context of Cochrane reviews, given they were unfamiliar to consumers in our study. This unfamiliarity has been found by others to result in scepticism about the trustworthiness of information based on Cochrane Reviews.^9^, ^49^ Additionally, presenting the evidence from systematic reviews is a science in itself. It requires a solid understanding of evidence‐based health care, the evolving body of health communication research, and information design principles that may be beyond those without specialist training.^53^ Better links between researchers and information providers could facilitate the integration of evidence from systematic reviews with other information that consumers need, and aid its dissemination.^54^

Subsequent to the IN‐DEEP study, researchers have experimented with new and interactive ways to present treatment benefits and risks, online.^32^ Recent examples include interactive Summary of Findings tables and plain language summaries,^14^ and the BMJ's consumer‐friendly rapid recommendations.^55^ While their graphical presentation would likely improve the presentation of benefits and harms used on our website, our results speak to the need for embedding such resources within the broader information and support needs of consumers. Notably, a recent randomised controlled trial of web‐based pictorial formats to present MS treatment information revealed that the animated graphical presentations of benefits were less well understood, compared with static presentations.^12^

Another relevant development is a broadening of systematic review approaches, including network meta‐analysis (where multiple interventions can be compared head‐to‐head),^56^ mixed‐method reviews (where quantitative data are synthesized with qualitative experience data),^57^ and reviews of observational data about side effects.^58^ These types of reviews open the door for evidence summaries to better meet consumer needs, by presenting empirically derived comparative effectiveness, longer‐term data on potential harms, and the lived experience of others.

We acknowledge a number of limitations. First is the small number of participants who completed the online survey, limiting application of the evaluation results to a broader population. The low response rate to the survey may be due, in part, to dissemination or recruitment shortcomings: We were reliant on our project partners for website promotion, and we elected to promote the website, rather than explicitly recruit people to complete the survey. Alternatively, those who did not find the website helpful may not have chosen to complete the survey, potentially biasing results towards more favourable responses. Second, it is unclear how well the website caters to those with low literacy levels. The educational background of consumers and family members involved in the development was high (mainly university educated), and we did not use a standardised tool (eg, Flesch‐Kincaid Grade) to assess the readability of the website.^59^ However, the fact that we received largely positive feedback from survey responses, including from those who did have limited levels of education, suggests we may have met, at least, some of the information needs of people with limited literacy. A final limitation was that the assessment of important concepts, such as understanding, were self‐reported, rather than objectively measured. A more objective measure of understanding is likely to have generated more modest results,^12^ although this was not within the scope of this project.

## 5 CONCLUSION

This paper describes an approach to developing evidence‐based treatment information that is underpinned by in‐depth understanding of the information needs of consumers. This understanding was facilitated by the research team's ongoing partnership with consumers, throughout the development of the Cochrane Review information summaries, and the website that hosts them. The evaluation indicates that some of the unmet treatment information needs of people with MS were met through this novel partnership approach. Equally, we have demonstrated that consumers seek more than just the information contained in Cochrane Reviews to help them understand and apply treatment information. Future projects that include partnership with consumers, as well as with peak health organisations and other treatment information providers, may offer opportunities to fulfil these additional consumer treatment information needs that extend beyond treatment risks and benefits.

## FUNDING

Financial support for this study was provided in part by a grant from Multiple Sclerosis Research Australia (Grant number 00025) and MS Australia ACT/NSW/VIC. Richard Osborne was funded, in part, by a National Health and Medical Research Council Senior Research Fellowship (APP1059122). Graziella Filippini, Paola Mosconi, and Cinzia Colombo were funded in part by the Italian Multiple Sclerosis Foundation (Grant number 2010/R/19). The funding agreement ensured the authors' independence in designing the study, interpreting the data, writing, and publishing the report.

## CONFLICT OF INTERESTS

The authors declared no conflict of interest with respect to the research, authorship, and/or publication of this article.

## AUTHOR CONTRIBUTIONS

Conceptualization: Sophie Hill, Michael Summers, Graziella Filippini, Richard Osborne, Sue Shapland

Formal analysis: Melanie Hawkins, Anneliese Synnot, Richard Osborne, Sophie Hill, Bronwen Merner

Funding acquisition: Sophie Hill, Michael Summers, Graziella Filippini, Richard Osborne, Sue Shapland

Investigation: Anneliese Synnot, Melanie Hawkins, Sophie Hill, Richard Osborne

Methodology: Anneliese Synnot, Melanie Hawkins, Michael Summers, Graziella Filippini, Richard H Osborne, Sue Shapland, Catherine Cherry, Rwth Stuckey, Catherine Milne, Paola Mosconi, Cinzia Colombo, and Sophie Hill

Project administration: Anneliese Synnot

Supervision: Sophie Hill

Writing – original draft: Anneliese Synnot, Melanie Hawkins, Sophie Hill, Bronwen Merner

Writing – review and editing: Anneliese Synnot, Melanie Hawkins, Bronwen Merner, Michael Summers, Graziella Filippini, Richard H Osborne, Sue Shapland, Catherine Cherry, Rwth Stuckey, Catherine Milne, Paola Mosconi, Cinzia Colombo, and Sophie Hill

## Supporting information

Supporting info item
