# Supplementary material for: Producing an evidence‐based treatment information website in partnership with people affected by multiple sclerosis
Source: Health Sci Rep. 2018 Mar 6;1(3):e24. doi: 10.1002/hsr2.24 (PMC6266475; doi:10.1002/hsr2.24)
Supplement: Supplementary file 1 — Supporting info item [file HSR2-1-e24-s001.docx]

**Supporting Information**

**S1. Website feedback survey**


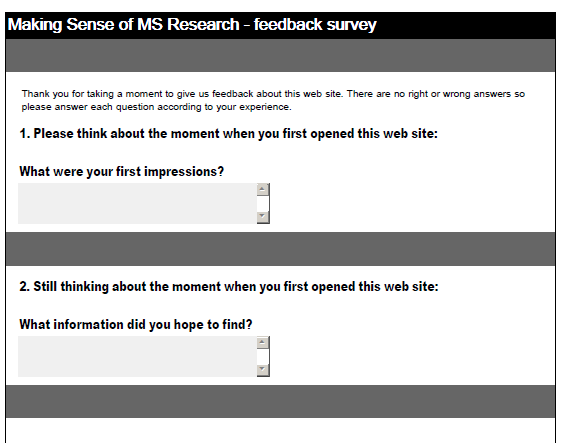


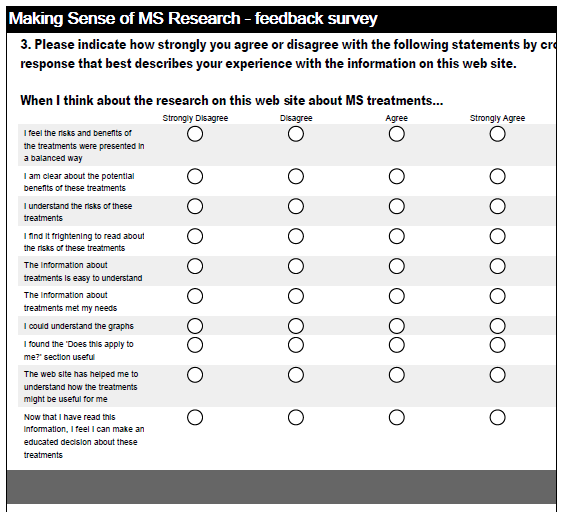


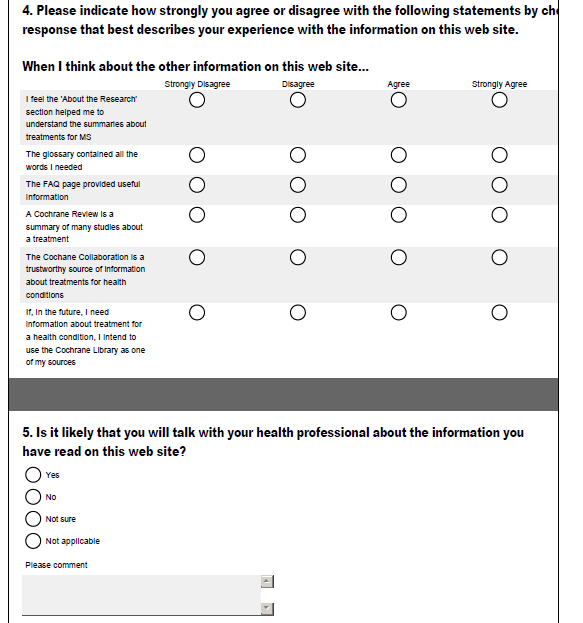


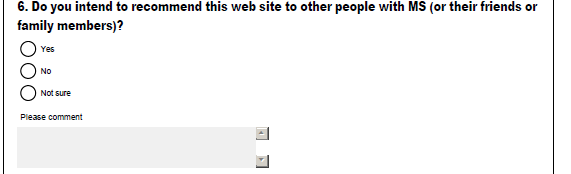


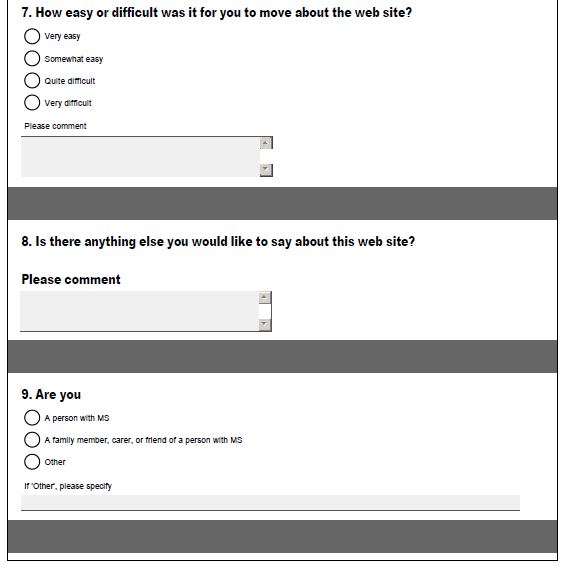


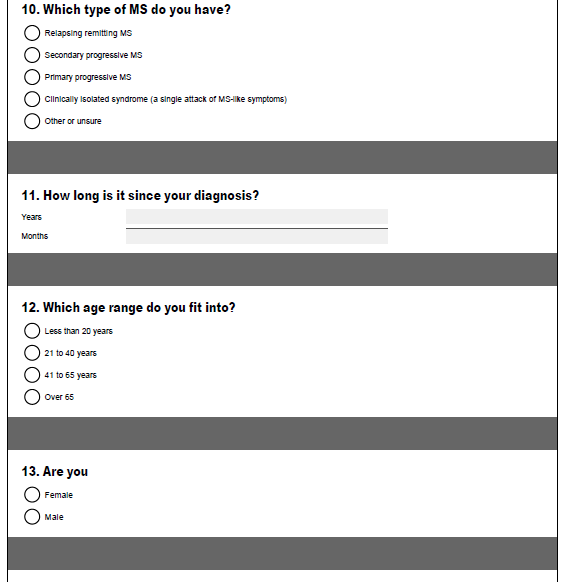


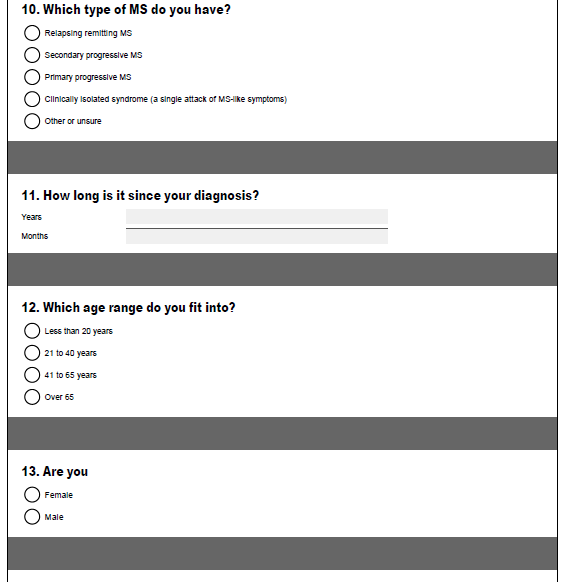


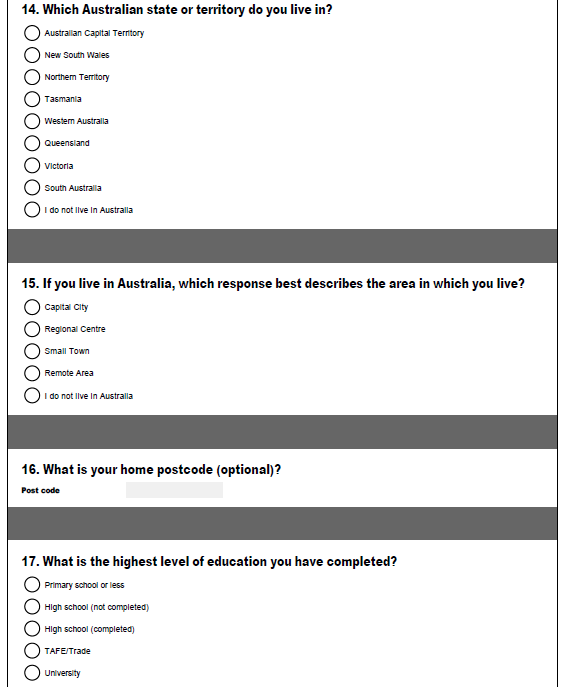


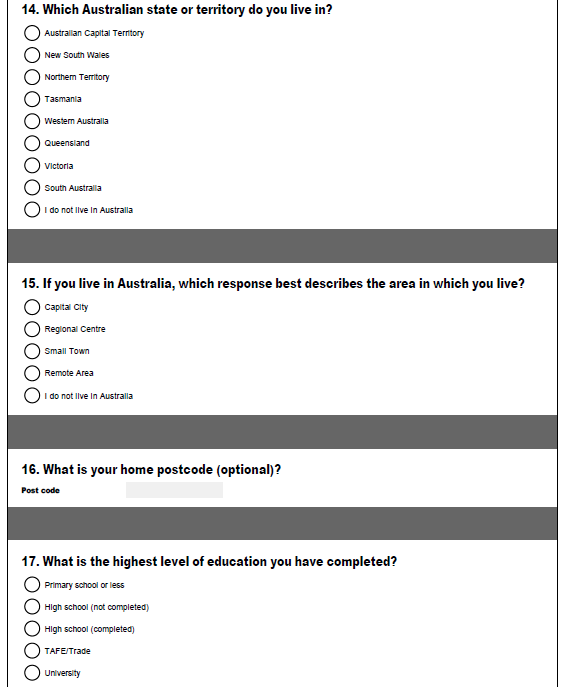


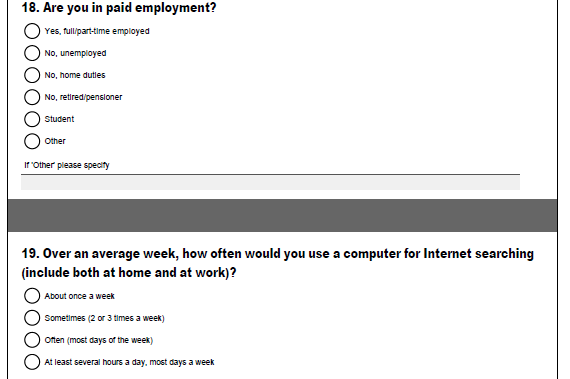


**S2 Table. Consumer evaluation of *[name anonymised]* website through online survey**

| **Online survey items** | **Response options** | | | | |
| --- | --- | --- | --- | --- | --- |
| **Survey item (n = 42, %)** | **SD** | **D** | **A** | **SA** | **NA** |
| I feel the risks and benefits of the treatments were presented in a balanced way | 2 (5) | 1 (2) | 28 (67) | 8 (19) | 3 (7) |
| I am clear about the potential benefits of these treatments | 2 (5) | 1 (2) | 29 (69) | 8 (19) | 2 (5) |
| I understand the risks of these treatments | 2 (5) | 2 (5) | 22 (52) | 14 (33) | 2 (5) |
| I find it frightening to read about the risks of these treatments^1^ | 11 (26)^1^ | 18 (43)^1^ | 6 (14)^1^ | 3 (7)^1^ | 4 (10) |
| The information about treatments is easy to understand | 2 (5) | 2 (5) | 19 (45) | 15 (38) | 3 (7) |
| The information about treatments met my needs | 4 (10) | 7 (17) | 22 (52) | 7 (17) | 2 (5) |
| I could understand the graphs | 2 (5) | 1 (2) | 21 (51) | 10 (24) | 8 (19) |
| I found the 'Does this apply to me?' section useful | 2 (5) | 5 (12) | 19 (45) | 9 (21) | 7 (17) |
| The website has helped me to understand how the treatments might be useful for me | 3 (7) | 5 (12) | 22 (52) | 8 (19) | 4 (10) |
| Now that I have read this information, I can make an educated decision about these treatments | 3 (7) | 6 (14) | 17 (40) | 11 (26) | 5 (12) |
| **Survey item (n = 36, %)** | **SD** | **D** | **A** | **SA** | **DK** |
| The ‘About the Research’ section helped me to understand the summaries about MS treatments | 1 (3) | 1 (3) | 23 (64) | 8 (22) | 3 (8) |
| The glossary contained all the words I needed | 1 (3) | 2 (6) | 21 (58) | 7 (19) | 5 (14) |
| The FAQ page provided useful information | 1 (3) | 3 (8) | 17 (47) | 7 (19) | 8 (22) |
| A Cochrane Review is a summary of many studies about a treatment | 1 (3) | 4 (11) | 15 (42) | 12 (33) | 4 (11) |
| The Cochrane Collaboration is a trustworthy source of information about treatments for health conditions | 1 (3) | 3 (8) | 16 (44) | 9 (25) | 7 (19) |
| If, in the future, I need information about treatment for a health condition, I intend to use the Cochrane Library as one of my sources | 2 (6) | 4 (11) | 16 (44) | 9 (25) | 5 (14) |
| **Survey item (n = 36, %)** |  | **Y** | **N** | **NS** | **NA** |
| Is it likely that you will talk with your health professional about the information you have read on this web site? | | 9 (25) | 9 (25) | 14 (39) | 4 (11) |
| **Survey item (n = 36, %)** | |  | **Y** | **N** | **NS** |
| Do you intend to recommend this web site to other people with MS (or their friends or family members)? | | | 24 (67) | 5 (14) | 7 (19) |
| **Survey item (n = 36, %)** | | **VE** | **SE** | **QD** | **VD** |
| How easy or difficult was it for you to move about the web site? | | 23 (64) | 10 (28) | 2 (6) | 1 (3) |

**Table 2 legend**

^1^This is a reversed scale so it means that 69% of the respondents *did not* find it frightening to read about the risks of these treatments.

Abbreviations: A = agree, D = disagree, DK = don’t know, N = no, NA = not applicable, NS = not sure, QE = quite difficult, SA = strongly agree, SD = strongly disagree, SE = somewhat easy, VE = very easy, VD = very difficult, Y = yes.
